# Robust charge-density wave strengthened by electron correlations in monolayer 1T-TaSe_2_ and 1T-NbSe_2_

**DOI:** 10.1038/s41467-021-26105-1

**Published:** 2021-10-07

**Authors:** Yuki Nakata, Katsuaki Sugawara, Ashish Chainani, Hirofumi Oka, Changhua Bao, Shaohua Zhou, Pei-Yu Chuang, Cheng-Maw Cheng, Tappei Kawakami, Yasuaki Saruta, Tomoteru Fukumura, Shuyun Zhou, Takashi Takahashi, Takafumi Sato

**Affiliations:** 1 Department of Physics, Graduate School of Science, Tohoku University, Sendai, 980-8578 Japan; 2 Center for Spintronics Research Network, Tohoku University, Sendai, 980-8577 Japan; 3 Advanced Institute for Materials Research (WPI-AIMR), Tohoku University, Sendai, 980-8577 Japan; 4 National Synchrotron Radiation Research Center, Hshinchu, 30077 Taiwan ROC; 5 State Key Laboratory of Low Dimensional Quantum Physics and Department of Physics, Tsinghua University, Beijing, 100084 China; 6 Department of Chemistry, Graduate School of Science, Tohoku University, Sendai, 980-8578 Japan; 7 Frontier Science Center for Quantum Information, Beijing, 100084 China

**Keywords:** Surfaces, interfaces and thin films, Electronic properties and materials

## Abstract

Combination of low-dimensionality and electron correlation is vital for exotic quantum phenomena such as the Mott-insulating phase and high-temperature superconductivity. Transition-metal dichalcogenide (TMD) 1T-TaS_2_ has evoked great interest owing to its unique nonmagnetic Mott-insulator nature coupled with a charge-density-wave (CDW). To functionalize such a complex phase, it is essential to enhance the CDW-Mott transition temperature *T*_CDW-Mott_, whereas this was difficult for bulk TMDs with *T*_CDW-Mott_ < 200 K. Here we report a strong-coupling 2D CDW-Mott phase with a transition temperature onset of ~530 K in monolayer 1T-TaSe_2_. Furthermore, the electron correlation derived lower Hubbard band survives under external perturbations such as carrier doping and photoexcitation, in contrast to the bulk counterpart. The enhanced Mott-Hubbard and CDW gaps for monolayer TaSe_2_ compared to NbSe_2_, originating in the lattice distortion assisted by strengthened correlations and disappearance of interlayer hopping, suggest stabilization of a likely nonmagnetic CDW-Mott insulator phase well above the room temperature. The present result lays the foundation for realizing monolayer CDW-Mott insulator based devices operating at room temperature.

## Abstract (web summary)

Monolayer transition metal dechalcogenides are a promising platform to study the interplay of low-dimensionality and electron correlations. Here, the authors report a Mott insulator state in monolayer 1T-TaSe_2_, that survives far above room temperature and is robust against external perturbations.

## Introduction

The interplay among electron correlation, dimensionality, and appearance of various quantum phases is a long-standing issue in condensed-matter physics. The correlated electron system is characterized by strong Coulomb interactions among electrons and the resultant emergence of exotic physical properties, which are absent in the weakly interacting electron system. The most drastic consequence of electron correlation is the typical Mott–Hubbard transition^1,2^ that converts a half-filled paramagnetic metal (predicted by single-particle theory) into an antiferromagnetic insulator when the on-site Coulomb interaction *U* exceeds the bandwidth *W* (i.e., effective Coulomb interaction *U*/*W* > 1). A more unusual phase is the nonmagnetic Mott insulator and associated exotic quantum phases, as highlighted by the quantum-spin-liquid phase in 1T-TaS_2_, a triangular lattice of two-dimensional (2D) Mott insulator with a CDW^3,4^. In comparison, the destruction of antiferromagnetic order in doped copper oxides leads to emergence of high-temperature superconductivity that coexists with charge order^5,6^.

To realize a Mott insulator, it is essential that the magnitude of *U*/*W* is above a critical value relevant to the structure and the electronic states of a material^1,2^. In fact, for an optimally doped copper oxide, the large *U* (~3 eV)^7^ estimated for Cu-3*d* electrons compared with the relatively small bandwidth *W* (~0.4 eV)^8^ also satisfies the condition of *U*/*W* ≫ 1. This suggests a direct relation between Mott physics and superconductivity (note that, in cuprates, the role of *U* is actually played by the charge-transfer gap of 1.4–2.0 eV^9^, but even in this case, the effective *U*/*W* (3.5–5.0) exceeds the critical value). However, the recent discovery of a Mott-insulator phase and associated superconductivity in tilted bilayer graphene^10,11^ demonstrated that even when *U* is considerably small (~30 meV), the band narrowing (*W* ~ 20 meV) introduced by the superstructure of moiré pattern can effectively convert a metallic state into a Mott-insulating one. This points to the importance of bandwidth control for materials with small *U* to trigger the Mott transition.

**Fig. 1:**
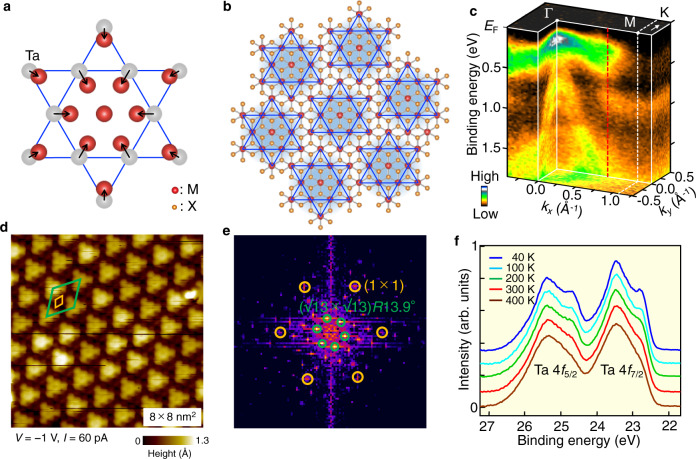
Schematics of star-of-David clusters and core-level photoemission spectrum of monolayer 1T-TaSe_2_. **a** Schematics of the displacement of Ta atoms in the star-of-David cluster. M and X represent transition-metal and chalcogen atoms, respectively. **b** Schematics of crystal structure for monolayer 1T-TaSe_2_ and star-of-David clusters with the √13 × √13 periodicity. **c** 3D ARPES-intensity plot as a function of 2D wave vector (*k*_*x*_ and *k*_*y*_) and *E*_B_ for monolayer 1T-TaSe_2_ measured at *T* = 40 K with the He-Iα line (*hν* = 21.218 eV). Hybridization gap (*k*_*x*_ ~ 2/3 ΓM) is indicated by red dashed line. **d** STM image in a surface area of 8 × 8 nm^2^ for monolayer 1T-TaSe_2_ on bilayer graphene measured at *T* = 4.8 K. **e** Fourier transform image of **d**. **f** Temperature dependence of EDC around the Ta-4*f* core level measured with *hν* = 260 eV for monolayer 1T-TaSe_2_.

The layered transition-metal dichalcogenide (TMD) 1T-TaS_2_ is believed to be a special example of a bandwidth-controlled Mott-transition material^12,13^ in the absence of magnetic order. Bulk 1T-TaS_2_ undergoes a Mott transition accompanying a commensurate charge-density wave (CDW) characterized by the star-of-David cluster (Fig. 1a) with a √13 × √13 periodicity (Fig. 1b), at *T*_CDW-Mott_ ~ 200 K. It is noted that twelve Ta atoms located at the corners of a cluster are slightly displaced from the original position toward the central Ta atom (Fig. 1a). 1T-TaS_2_ satisfies the half-filling condition necessary for realizing a Mott-insulator phase, since 12 electrons at the displaced 12 Ta atoms form the fully occupied 6 bands and the remaining electron at the central Ta atom forms a half-filled metallic band^14,15^. Although the *U* of Ta 5*d* electrons is relatively small (~0.7 eV)^16^, 1T-TaS_2_ undergoes the Mott transition when the half-filled band is narrowed to the scale of *U* due to the band folding associated with the CDW^12,13^ in a similar manner to tilted bilayer graphene. More interestingly, it was shown that while it has a charge gap of ~0.3 eV, it shows gapless quantum-spin-liquid dynamics and no long-range magnetic order down to 70 mK^17^. Recently, the exploration for Mott phases coexisting with CDW was extended to the atomic-layer limit in TMDs as in graphite (graphene), with the possible emergence of exotic quantum phenomena in the pure 2D limit^18–20^. However, the nature of a pure 2D CDW-Mott phase, such as its robustness, possibility for magnetism, and differences if any, compared with the 3D bulk case, has been scarcely explored experimentally. In particular, the essential issue regarding the interplay between the Mott phase and dimensionality is yet to be clarified.

In this work, we address all the above key issues by performing a comprehensive angle-resolved photoemission spectroscopy (ARPES) study on epitaxially grown monolayer 1T-TaSe_2_ and 1T-NbSe_2_, and demonstrate the robust CDW-Mott phase under external perturbations such as heating and electron doping.

## Results and discussion

### Characterization of TaSe_2_

First, we discuss the electronic structure of monolayer 1T-TaSe_2_ whose monolayer nature was confirmed by our scanning tunneling microscopy (STM) measurement (Supplementary note 1). Figure 1c displays the 3D ARPES intensity plotted as a function of 2D wave vector (*k*_*x*_ and *k*_*y*_) and binding energy *E*_B_ measured at *T* = 40 K. One can clearly recognize a nearly flat band at *E*_B_ ~ 0.3 eV and dispersive holelike bands topped at the Γ point, which are ascribed to the Ta 5*d* and Se 4*p* bands, respectively^18^. The topmost Ta 5*d* band does not cross the Fermi level (*E*_F_) and exhibits an insulating gap of ~0.3 eV below *E*_F_ at the Γ point. This gap is not assigned to a band gap, a substrate-induced gap, or a conventional CDW gap (Supplementary note 2), but to a Mott–Hubbard gap. This Mott gap is associated with the enhancement of *U*/*W* caused by the hybridization of backfolded bands and the resultant band narrowing due to the √13 × √13 commensurate CDW (Fig. 1b), as in bulk nonmagnetic 1T-TaS_2_^12,13^ and a surface layer of bulk 1T-TaSe_2_^21,22^ as can be suggested from the overall similarity of experimental band dispersion (Supplementary Fig. S2). The gap size below *E*_F_, called here Δ_Mott_, roughly corresponds to a half of the full Mott-gap size 2Δ_Mott_ because *E*_F_ is nearly located at the midpoint between the lower Hubbard band (LHB) and the upper Hubbard band (UHB) as suggested from the comparison of ARPES and tunneling spectroscopy data^20,23,24^ (Supplementary note 3). As shown in Fig. 1c, a signature of the CDW is clearly seen as an apparent hybridization-gap discontinuity^21,22^ in the band dispersion at *k* ~ 2/3 ΓM (red dashed line, see Fig. 1c). It is important to note that we could selectively fabricate a pure 1T-TaSe_2_ phase (and also 1T-NbSe_2_ phase, discussed later) with ease by controlling the substrate temperature^18^. This enables observation of a clear hybridization-gap discontinuity in our data as compared with a recent study, where admixture from the 1H-TaSe_2_ phase made it difficult to see the discontinuity^20^. The STM image in Fig. 1d obtained in a spatial region of 8 × 8 nm^2^ on a monolayer TaSe_2_ island signifies a clear periodic modulation associated with the formation of CDW containing the hexagonal lattice of star-of-David clusters. We have confirmed that this lattice has a periodicity of √13 × √13*R*13.9° expected for the formation of star-of-David lattice, as well visible as superspots in the Fourier transform image shown in Fig. 1e, in agreement with the previous STM study of monolayer 1T-TaSe_2_^20^.

The Ta 4 *f* core-level spectroscopy (Fig. 1f) signifies that the Ta 4*f*_5/2_ and 4*f*_7/2_ spin-orbit satellite peaks split into two subpeaks, as is clearly visible in the energy-distribution curve (EDC) at *T* = 40 K. Since the additional splitting of Ta-4*f* peak is attributed to the different electron density at each Ta site^25,26^ and/or the change in the chemical bonding of Ta atoms due to the formation of the star-of-David clusters, the core-level spectrum is consistent with our STM data that support the formation of the star-of-David clusters. On elevating temperature, we found that the lower-binding-energy subpeak of both the Ta4*f*_5/2_ and 4*f*_7/2_ components is gradually weakened, but the shoulder feature still remains even at *T* = 400 K. This implies that the Mott phase survives much above the room temperature. We will come back to this point later.

### Temperature dependence of the Mott gap

**Fig. 2:**
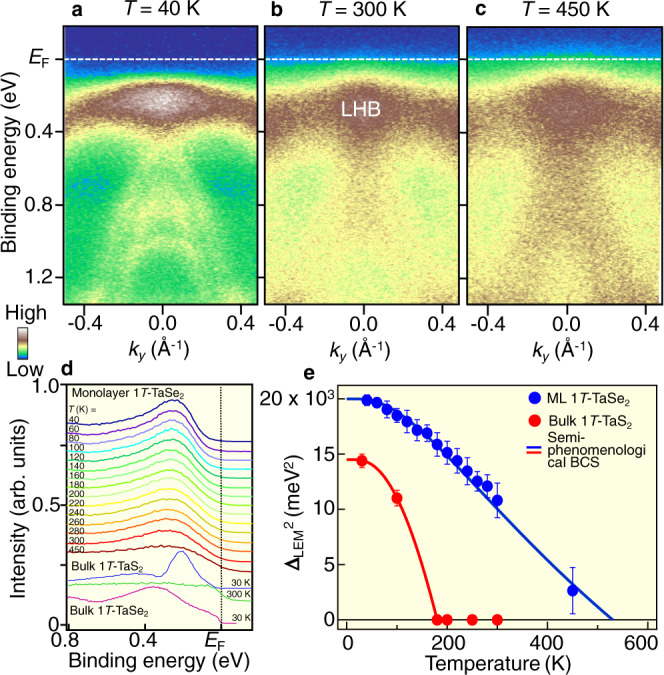
CDW–Mott phase of monolayer 1T-TaSe_2_ robust against temperature variation. **a**-**c** Near-*E*_F_ ARPES intensity along the ΓK cut measured at *T* = 40, 300, and 450 K, respectively. **d** Temperature dependence of EDC at the Γ point. EDC for bulk 1T-TaS_2_ (*T* = 30 and 300 K) and bulk 1T-TaSe_2_ (*T* = 30 K) is also shown as a reference. **e** Squared leading-edge midpoint Δ_LEM_ at the Γ point plotted against *T* for monolayer 1T-TaSe_2_ (blue circles), together with the numerical fitting results with the semiphenomenological (blue solid curve) BCS gap functions. Δ_LEM_ and fitting results are also plotted for bulk 1T-TaS_2_ (red). We have obtained {*T*_CDW-Mott_, *A*} = {530 K, 1.01} and {180 K, 1.50} for monolayer 1T-TaSe_2_ and bulk 1T-TaS_2_, respectively. Error bars in **e** reflect the uncertainties originating from the energy resolution and the standard deviation in the peak positions of EDCs.

The formation of CDW is further corroborated by the appearance of a LHB in the ARPES intensity at *T* = 300 K (Fig. 2b), similarly to the case at *T* = 40 K (Fig. 2a), because the Mott gap cannot be formed without the CDW^12,13^. Intriguingly, the LHB survives even upto *T* = 450 K (the highest temperature in our experimental setup, see Fig. 2c), whereas the overall spectral feature becomes less clear. Such spectral feature at *T* = 450 K cannot be explained in terms of the absence of Mott gap and the persistence of CDW gap because of the following reason. In bulk TaSe_2_^27^, the LHB essentially vanishes at room temperature and a large metallic spectral weight emerges at *E*_F_, in contrast to the low-temperature (70–220 K) data that display a peak associated with the LHB. Our ARPES data for monolayer 1T-TaSe_2_ at room temperature resemble that of bulk TaSe_2_ at low temperature (Fig. 2d), suggestive of the persistence of a Mott gap at *T* = 450 K (Supplementary note 4). The robustness of Mott gap is also seen from the detailed temperature dependence of EDC at the Γ point in Fig. 2d. This is in stark contrast to bulk 1T-TaS_2_ where a metallic Fermi edge is recovered at *T* = 300 K. Also, this is distinct from bulk 1T-TaSe_2_ that shows a clear Fermi-edge cutoff even at *T* = 30 K (Fig. 2d) and hence, we compared the *T*-dependent behavior of the clear gap observed in monolayer 1T-TaSe_2_ with bulk 1T-TaS_2_.

To discuss more quantitatively the gap behavior, we plot the binding energy of the leading-edge midpoint of the EDC, Δ_LEM_, as a function of temperature for monolayer 1T-TaSe_2_, and compare it with that for bulk 1T-TaS_2_. We expect Δ_LEM_ to be directly related with the transport gap in the monolayer instead of the spectroscopic gap Δ_Mott_ (for the difference between Δ_LEM_ and Δ_Mott_, see Supplementary note 5). As one can see from the Δ_LEM_^2^ vs *T* plot in Fig. 2e, Δ_LEM_^2^ for monolayer 1T-TaSe_2_ shows a nearly linear behavior as a function of *T* near *T*_CDW-Mott_, and exhibits a finite value even at 450 K. This nearly linear behavior is also seen in bulk 1T-TaS_2_ as shown in Fig. 2e, and was also reported for bulk and monolayer 1T-TiSe_2_ recently^28^. The *T* dependence of Δ_LEM_ is well reproduced by a semiphenomenological BCS gap equation based on the mean-field approximation (blue solid curve) expressed as Δ_LEM_(*T*)^2^−Δ_LEM_ (*T*_CDW-Mott_)^2^ ∝ tanh^2^ (*A*√((*T*_CDW-Mott_/*T*)−1)), where Δ_LEM_, *T*_CDW-Mott_, and A are the binding energy of the leading-edge midpoint, the CDW–Mott transition temperature, and the proportional constant, respectively^28^, which was recently used to characterize bulk and monolayer 1T-TiSe_2_ (note that the observed temperature dependence of EDC can hardly be explained with the thermal broadening; for details, see Supplementary note 4). From the numerical fittings, the transition temperature was estimated to be *T*_CDW-Mott_ ~ 530 K for monolayer 1T-TaSe_2_, and this is much higher than that obtained for bulk 1T-TaS_2_ (<200 K; red circles and curves). The present results suggest that the CDW-Mott-transition temperature *T*_CDW-Mott_ of monolayer TaSe_2_ is very high, being drastically higher than that of bulk TaS_2_ (*T*_CDW-Mott_ ~ 200 K)^12^ and a surface layer of bulk TaSe_2_ (*T*_CDW-Mott_ ~ 260 K)^27,29^ (note that a consensus has not been reached for the exact *T*_CDW-Mott_ value at the surface of TaSe_2_; Supplementary note 6 and Fig. S5). In contrast, the increase in *T*_CDW_ of 1T-TiSe_2_ in going from bulk (*T*_CDW_ ~ 200 K) to monolayer (*T*_CDW_ ~ 235 K) was small^28,30^. If we consider Δ_LEM_ to be a measure of half the transport gap (since *E*_F_ lies in the middle of the gap, as discussed earlier), 2Δ_LEM_(*T* = 0)/*k*_B_*T*_CDW-Mott_ ~ 12, which is significantly larger than the weak-coupling result of ~3.52. Thus, monolayer 1T-TaSe_2_ can be classified as a strongly coupled CDW–Mott phase. It is noted that our time-resolved ARPES experiment suggests that the LHB of monolayer TaSe_2_ survived after photoexcitation even when we adopted the maximum pump fluence above which monolayer samples were damaged (this maximum pump fluence is lower than that in bulk TaS_2_;^31–36^ for details, see Supplementary note 7 and Fig. S6).

### Carrier doping effect to the Mott gap

**Fig. 3:**
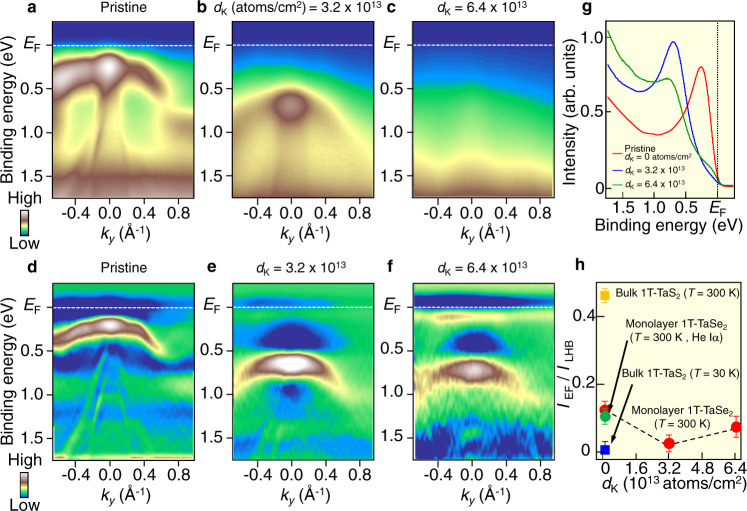
CDW–Mott insulator phase in monolayer 1T-TaSe_2_ robust against electron doping. **a**–**c** K-deposition dependence of ARPES intensity along the ΓK cut for monolayer 1T-TaSe_2_ [potassium coverage *d*_K_ = 0 (pristine), 3.2 × 10^13^, and 6.4 × 10^13^ atoms/cm^2^, respectively], measured at *T* = 300 K with *hν* = 51 eV. **d**–**f** Same as **a**–**c**, but obtained by taking the second derivative of EDCs. **g** EDCs at the Γ point for each *d*_K_. **h** Plots of intensity at *E*_F_ with respect to that at LHB, *I*_EF_/*I*_LHB_, as a function of *d*_K_, estimated from the EDCs in **g**. The values for bulk TaS_2_ measured at *T* = 30 and 300 K are also plotted. Error bars reflect the uncertainties originating from the energy resolution and statistics of data. Asymmetry in the intensity profile in **a** and **b** is associated with the inequivalent photoelectron matrix-element effect between positive and negative *k*_*y*_’s (Supplementary note 8 and Fig. S7).

Next, we show the robustness of the LHB against carrier doping. Figures 3a–c show the evolution of ARPES intensity as a function of potassium (K) coverage *d*_K_ [0 (pristine), 3.2 and 6.4 × 10^13^ atoms/cm^2^] and the corresponding second-derivative intensity plots (d–f) (for details of the *d*_K_ estimation, see Methods section). Upon K deposition of *d*_K_ = 3.2 × 10^13^ atoms/cm^2^, which corresponds to ~50% of the star-of-David density (Fig. 3b), the band structure is shifted downward as a whole with respect to pristine TaSe_2_ (Fig. 3a) due to the electron doping from K atoms. This suggests that the K deposition dopes electron carriers into a whole monolayer film, as in the case of other monolayer TMD films such as TiSe_2_ where K deposition causes an overall downward shift of the band structure and disappearance of CDW^37^. On further depositing K atoms (Fig. 3c), the spectral feature becomes significantly broad due to the strong electron scattering by the K-induced disorder, whereas a broad hump originating from the LHB is still seen at *E*_B_ ~ 0.6 eV in the EDC in Fig. 3g. The LHB and its systematic downward shift are better visualized in the second-derivative-intensity plots in Fig. 3d–f. A careful look at the intensity for *d*_K_ = 6.4 × 10^13^ atoms/cm^2^ (Fig. 3f) reveals a bright intensity in the vicinity of *E*_F_, which originates from a finite Fermi-edge cutoff, as also seen in the EDC (green curve) in Fig. 3g.

To discuss the spectral evolution upon K dosing in more detail, we have analyzed the spectral weight at *E*_F_ relative to that of LHB, *I*_EF_/*I*_LHB_, as a function of K coverage *d*_K_. As shown in Fig. 3h, the *I*_EF_/*I*_LHB_ value (red circles) does not exhibit a monotonic behavior as a function of *d*_K_, showing a minimum at *d*_K_ = 3.2 × 10^13^ atoms/cm^2^. The nonzero value for *d*_K_ = 0 atoms/cm^2^ may be associated with the tail of LHB extending toward *E*_F_, as also seen in the EDC (Fig. 3g). We found that the *I*_EF_/*I*_LHB_ value in monolayer for both *d*_K_ = 3.2 and 6.4 × 10^13^ atoms/cm^2^ is larger than that of the CDW–Mott insulator phase (*T* = 30 K) in bulk TaS_2_ (blue square), implying a possible metallic behavior. We have confirmed that such a difference between monolayer and bulk is not associated with the difference in the experimental conditions. This can be seen from Fig. 3h where *I*_EF_/*I*_LHB_ at *T* = 300 K for monolayer 1T-TaSe_2_ (*d*_K_ = 0) obtained with He lamp (green circle) and synchrotron radiation (red circle) well coincide with each other within our experimental uncertainty.

A simple explanation to account for the observed metallic component may be electron occupation of the UHB. Since the energy position of LHB shifts from 0.28 eV to 0.75 eV with K deposition (Fig. 3g), one would expect the UHB to appear below *E*_F_ in the *d*_K_ = 6.4 × 10^13^ atoms/cm^2^ sample since the full Mott gap is estimated to be 0.5 eV (Supplementary note 3). However, we found no evidence for the prominent peak from the UHB. This is reasonable because the high Coulomb cost *U* to populate an electron to the UHB does not guarantee the rigid-band-like electron doping. As an alternative possibility, a metallic K component due to the high density of K atoms (which produces an angle-integrated-type background with a weak Fermi-edge cutoff in EDC) or in-gap states (with suppressed quasiparticle intensity and reduced lifetime) could be conceived. The latter possibility is expected from the Hubbard model for the doped Mott insulator and was observed in spectroscopic studies of cuprates^38^.

As shown in Fig. 3h, the *I*_EF_/*I*_LHB_ value in the monolayer sample of *d*_K_ = 6.4 × 10^13^ atoms/cm^2^ is much smaller than that in the bulk counterpart with the fully melted CDW–Mott state at *T* = 300 K (yellow square). This implies that the framework of LHB itself is still maintained in the monolayer even when the system likely becomes metallic upon electron doping, in contrast to the bulk counterpart where even a small amount of electron doping breaks the LHB and leads to the occurrence of superconductivity^39–42^. In particular, doping electrons by substitution of magnetic Fe ions in 1T-Fe_*x*_Ta_1−*x*_S_2_ was shown to result in a dispersive electron band accompanied by a destruction of the LHB even with 1% Fe substitution^40,41^. A plausible mechanism of such fragileness in bulk is associated with the Fermi-surface nesting condition that is sensitive to the carrier doping, and has been discussed as a primary cause of incommensurate CDW and the resultant Mott phase in bulk 1T-TaSe_2_^43,44^. In this regard, the monolayer data are puzzling and surprising because the LHB still survives even when the nesting condition is modified by the electron doping. In any case, the survival of LHB suggests that the electron correlation is still strong even in the doped monolayer sample.

A recent generalized-gradient approximation band-structure calculation with on-site Coulomb interaction (GGA + U) for isoelectronic 1T-NbSe_2_^45^ has reproduced the LHB and Mott gap, consistent with the ARPES data. It is noted though, while the GGA + U study and a very recent DFT (density-function-theory) calculation with GGA on monolayer 1T-TaSe_2_^46^ suggested a spin-1/2 ferromagnetic Mott-insulator phase, our experimental attempt to detect possible ferromagnetism was not successful [since the detection of ferromagnetism by macroscopic magnetization measurement is difficult for monolayer samples, we carried out a very primitive experiment by just putting a strong Nd magnet (magnetic field ~ 500 mT) on top of a film to detect possible attractive force]. A good agreement of the overall band structure in the Mott-insulator phase between monolayer TaSe_2_ and bulk nonmagnetic 1T-TaS_2_^41^ may support the nonmagnetic ground state of monolayer TaSe_2_, although this point needs to be verified in future, e.g., by X-ray magnetic circular dichroism measurement.

### Comparison between TaSe_2_ and NbSe_2_

**Fig. 4:**
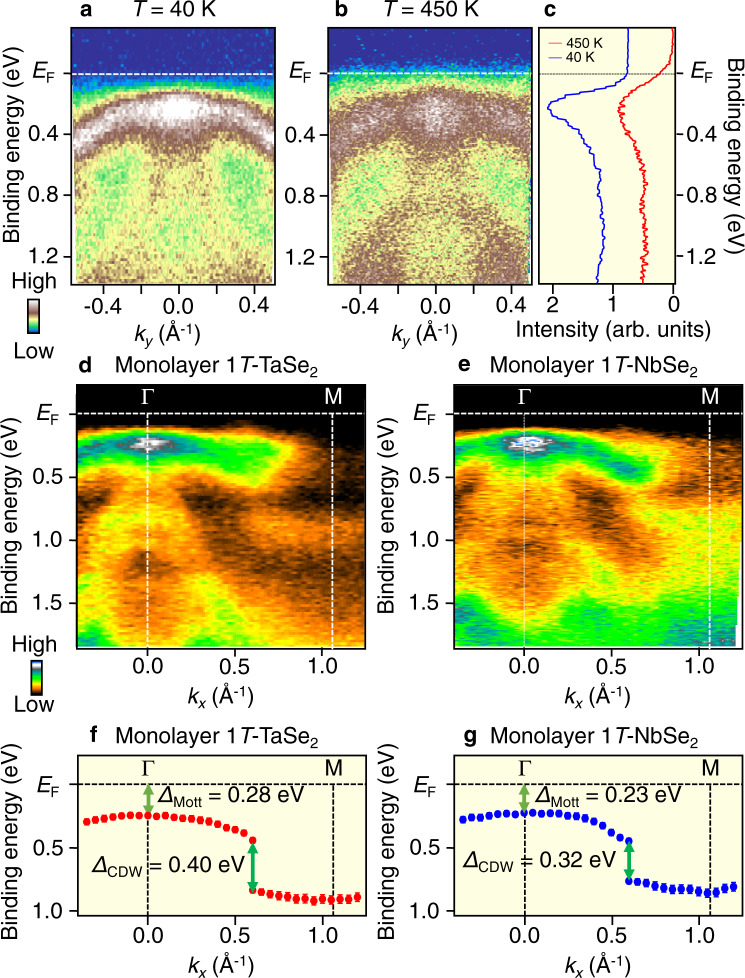
Comparison of the CDW–Mott phase between monolayer NbSe_2_ and TaSe_2_. **a**, **b** ARPES intensity of monolayer 1T-NbSe_2_ at *T* = 40 and 450 K, respectively, measured along the ΓK cut. **c** EDCs at the Γ point at *T* = 40 and 450 K. **d**, **e** Valence-band ARPES intensity along the ΓM cut for monolayer NbSe_2_ and TaSe_2_, respectively. **f**, **g** Experimental band dispersion extracted by tracing the peak position in EDCs for monolayer TaSe_2_ and NbSe_2_, respectively, highlighting the quantitative difference in the magnitude of the Mott–Hubbard gap (Δ_Mott_) and the hybridization gap associated with CDW (Δ_CDW_). Error bars in **e** reflect the uncertainties originating from the energy resolution and the standard deviation in the peak positions of EDCs.

Now that the survival of LHB in various conditions is established for monolayer TaSe_2_, next, we explore the CDW–Mott phase in a cousin material, monolayer 1T-NbSe_2_. One can immediately recognize in the ARPES intensity along the ΓK cut in Fig. 4a that the LHB is well seen at *T* = 40 K in monolayer NbSe_2_. The LHB survives even at *T* = 450 K (Fig. 4b), as is also visible in the EDC at the Γ point in Fig. 4c. A side-by-side comparison of the valence-band ARPES intensity along the ΓM cut between monolayer TaSe_2_ and NbSe_2_ in Fig. 4d, e reveals several common features, such as a nearly flat LHB, dispersive hole-like Se-4*p* bands, and a discontinuity of band dispersion at *k*_*x*_ ~ 2/3 ΓM caused by the hybridization-gap opening due to the CDW. These results demonstrate that the robust Mott-insulator phase coexisting with CDW upon heating is a common characteristic for monolayer TaSe_2_ and NbSe_2_.

In the following, we discuss why the LHB in monolayer TaSe_2_ and NbSe_2_ is robust unlike in the bulk counterpart. Since one of the key parameters to trigger a CDW–Mott transition is the effective Coulomb interaction *U*/*W*, it is important to discuss the independent roles of how *U* and *W* are independently affected on going from the bulk 3D structure to the monolayer 2D case. The effective on-site Coulomb correlation energy can be described by the equation *U* = *E*_I_ – *E*_A_ – *E*_Pol_ where, *E*_I_ is the ionization energy, *E*_A_ is the electron affinity, and *E*_Pol_ is the polarization energy that arises from screening due to any electronic perturbation such as removing or adding an electron. This screening causes a strong reduction of *U* compared with the bare Coulomb interaction *U*_bare_ (= *E*_I_ – *E*_A_). For example, based on a one-band Hubbard model, *U* for the Cu site gets reduced to ~4 eV in La_2_CuO_4_ compared with *U*_bare_ ~20 eV for Cu atom^47^. For 4*d* and 5*d* transition metals, *U* is expected to be still lower. For 1T-TaS_2_, 1T-TaSe_2_, and 1T-NbSe_2_, typical values of *U* reported in the literature range from ~0.4/0.7 eV (in the GW approximation/DFT-DMFT approximation^16,48^) to ~2.0/2.8 eV (in GGA + *U*)^20,49,50^. Considering the role of screening in monolayer compared to the bulk case, while the intralayer *E*_Pol_ is expected to show negligible changes in the monolayer case, the interlayer *E*_Pol_ would be suppressed as there are no other layers and the interaction with the substrate is weak, resulting in an effective increase in *U* compared with the bulk.

**Fig. 5:**
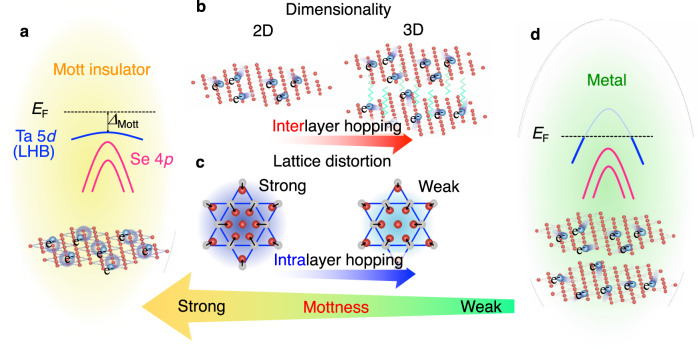
Realization of 2D CDW–Mott insulator phase assisted by strong lattice distortion. **a** Schematics of (top) the band dispersion and (bottom) the star-of-David clusters in the robust CDW–Mott phase in monolayer TaSe_2_. **b** Illustration to compare the interlayer hopping in the crystal in 2D and 3D systems. **c** Illustration of strong vs weak lattice distortion in the star-of-David cluster and its relationship with the intralayer hopping in the CDW phase. **d** Schematics of (top) the normal-state band dispersion and (bottom) the bulk crystal structure without CDW formation.

Similarly, since there is no out-of-plane or interlayer hopping in the monolayer, i.e., the intrinsic bulk interlayer bandwidth *W*_out_ is absent, the net effective bandwidth *W* will get reduced. It was suggested from the first-principles band-structure calculations that, although the in-plane bandwidth *W*_in_ becomes significantly small (~0.08 eV^51^) due to the band reconstruction associated with CDW, the out-of-plane bandwidth *W*_out_ (~0.54 eV^51^) does not suffer from a strong band-narrowing effect because of the in-plane nature of CDW. In this case, the dominant channel to determine the total *W* is the interlayer hopping (Fig. 5b) (note that the inter- and intralayer hopping channels do not contribute in an additive way to the bandwidth, but one can still discuss which plays a dominant role). It is thus inferred that the *U*/*W* value in the bulk is largely governed by the interlayer hopping and the bulk is located on the verge of the CDW–Mott transition (*U*/*W* ~ 1.3;^48^ note that *U* is ~0.7 eV in TaS_2_^16^). On the other hand, in monolayer, the interlayer hopping is intrinsically absent (Fig. 5b) and the net *W* is simply associated with the intralayer hopping. Thus, both the increase in *U* and decrease in *W* are expected to positively work together to efficiently increase *U*/*W*, leading to the robust CDW–Mott phase far above bulk *T*_Mott_. We remark here that it is difficult to experimentally determine *W* by simply tracing the continuously dispersing bands in the experiment, because such bands are composed of multiple subbands reconstructed by the CDW and the intensity of bands is often suppressed in the region away from the original unfolded band^33^. The Mott transition is associated only with a half-filled subband crossing *E*_F_, which has a narrow in-plane bandwidth *W*_in_^33^, although previous ARPES studies above *T*_Mott_ on bulk TaS_2_ (e.g.,^21,26,27,41^) were unable to resolve this band, probably because of the smearing of fine-band structure by, e.g., thermal broadening.

A comparison of characteristic energy scales between monolayer TaSe_2_ and NbSe_2_ reveals an intriguing aspect of the CDW–Mott phase in the two systems (Fig. 4d and e). As shown in the ARPES-derived band dispersions in Fig. 4f and g, a half of the full Mott gap, Δ_Mott_, in TaSe_2_ (0.28 ± 0.02 eV) is slightly larger than that in NbSe_2_ (0.23 ± 0.02 eV). Also, the hybridization gap Δ_CDW_ in TaSe_2_ (0.40 ± 0.03 eV) is larger than that in NbSe_2_ (0.32 ± 0.03 eV). According to the general trend of *U* in 3*d*–4*d*–5*d* electron systems, *U* for the Nb–4*d* orbital is expected to be larger than that for the Ta-5*d* orbital. Band-structure calculations suggested that the in-plane bandwidth of Nb 4*d* band in the normal state of monolayer NbSe_2_ (~2.2 eV)^49^ is smaller than that in monolayer TaSe_2_ (~2.7 eV)^52^. It is expected from a simple band-folding picture that the bandwidth in the CDW phase *W*_in_ is also smaller in NbSe_2_. Although this argument suggests a larger *U*/*W* and a resultant more stable CDW–Mott phase in NbSe_2_ than in TaSe_2_, the observed smaller Δ_Mott_ (0.23 eV) in NbSe_2_ (this also implies lower *Τ*_Mott_) apparently contradicts with the above simple argument. This discrepancy may be reconciled by taking into account the observed larger Δ_CDW_ (0.40 eV) in TaSe_2_, which suggests a smaller intralayer hopping and as a result a larger *U*/*W* compared with NbSe_2_ (note that while the bandwidth of LHB is estimated to be 0.19 and 0.21 eV for TaSe_2_ and NbSe_2_, respectively, these values cannot be regarded as *W* and one needs to know the original bare *W* without any influence from the Mott gap). This suggests that the lattice displacement in the star-of-David cluster is stronger in monolayer TaSe_2_ (Fig. 5c; left), which is also inferred from the stronger metallic-bonding character of Ta than that of Nb. The stronger periodic lattice distortion due to the CDW in TaSe_2_ is also supported by the observation of more pronounced hybridization-gap discontinuity around *k* ~ 2/3 ΓM in TaSe_2_, as seen from the stronger intensity suppression at *E*_B_ ~ 0.6 eV in Fig. 4d than that in Fig. 4e (note that some other experiments must be carried out to firmly establish the stronger CDW in TaSe_2_). It is also noted that Δ_CDW_ of monolayer TaSe_2_ (0.40 eV) is slightly larger than that of bulk TaSe_2_ (0.37 eV);^22^ this suggests a stronger lattice distortion in monolayer systems, consistent with GGA + U calculations discussed above^45^. All these arguments suggest that the robust CDW–Mott insulator phase of monolayer TaSe_2_ and NbSe_2_ (Fig. 5a) is caused by the disappearance of interlayer hopping assisted by a strong lattice distortion. In other words, the robust CDW in monolayer TaSe_2_ and NbSe_2_ is derived from a combination of electron correlations, a strong lattice distortion, and the absence of interlayer hopping. It is emphasized that such properties are all linked to the electron hopping (or electron kinetic energy) of the system (Fig. 5b and c), and the controllability of the Mottness (i.e., strength of the Mott phase) lies on how to effectively manipulate both inter- and intralayer hopping (Fig. 5a–d).

The present study has established an effective means to stabilize the CDW–Mott phase in terms of band engineering. Also, the discovery of the robust CDW–Mott phase far above the room temperature is useful for developing practical CDW–Mott insulator-based ultrathin nanoelectronic devices. It would be very interesting to explore the superconductivity in a metallic state near the Mott phase.

## Methods

### Sample preparation

Monolayer 1T-TaSe_2_ and 1T-NbSe_2_ films were grown on bilayer graphene/6H-SiC by using molecular-beam-epitaxy (MBE) method in an ultrahigh vacuum (UHV) of 3 × 10^-10^ Torr. As for the monolayer NbSe_2_ film, we have adopted the same procedure to grow monolayer 1T-NbSe_2_ established in our previous studies where the 1T structure, √13 × √13 lattice reconstruction, and its monolayer nature were already clarified^19,53^. To fabricate a monolayer TaSe_2_ film, we have also followed the fabrication method established by ourselves^18^. Specifically, bilayer graphene was prepared by annealing an *n*-type Si-rich 6*H*-SiC(0001) single-crystal wafer, with resistive heating at 1100 °C in ultrahigh vacuum better than 1 × 10^−10^ Torr for 30 min. A monolayer TaSe_2_ (NbSe_2_) film was grown by evaporating Ta (Nb) on the bilayer graphene substrate kept at 560 °C (580 °C) under a Se atmosphere^18,19^. The as-grown film was subsequently annealed at 400 °C for 30 min. The growth process was monitored by reflection high-energy reflection diffraction (RHEED). The film thickness was estimated by a quartz-oscillator thickness meter, scanning tunneling microscopy (STM), and atomic force microscopy (AFM). Based on our experience of fabricating various monolayer TMD films such as NbSe_2_, VTe_2_, VSe_2_, and TiSe_2_^18,19,54,55^, a monolayer film is formed immediately after the disappearance of buffer-layer-originated 6√3 × 6√3 RHEED pattern upon coevaporation of transition-metal and chalcogen atoms. We have judged the thickness of 1T-TaSe_2_ and NbSe_2_ films by monitoring this disappearance in the RHEED pattern. After the fabrication by the MBE method, the films were transferred to the ARPES-measurement chamber without breaking the vacuum. We have calibrated the deposition rate of K atoms by calculating the volume of *π*-band Fermi surface at the K point in bilayer graphene on SiC with keeping the same evaporation rate as that in the case of monolayer TaSe_2_, and it is estimated to be 1.6 × 10^13^ atoms/cm^2^/min. We have deposited K atoms for 2 and 4 min. that corresponds to the K coverage *d*_K_ of 3.2 and 6.4 × 10^13^ atoms/cm^2^, i.e., ~50 and ~100% of the star-of-David density, respectively. Thus, the amount of K dosing with respect to the star-of-David density is sufficient to achieve a sizable electron doping into monolayer 1T-TaSe_2_.

### ARPES and STM measurements

ARPES measurements were carried out using an MBS-A1 electron-energy analyzer with a high-flux helium-discharge lamp and a toroidal grating monochromator at Tohoku University and an Omicron-Scienta R4000 electron-energy analyzer with synchrotron radiation at Taiwan Light Source (TLS), National Synchrotron Radiation Research Center (NSRRC). The energy and angular resolutions were set to be 12.5–40 meV and 0.2°, respectively. Core-level photoemission-spectroscopy measurement was performed at BL28A with microfocused beam spot in Photon Factory. Time-resolved ARPES measurements were carried out at Tsinghua University using an Omicron-Scienta DA30-L-8000 electron-energy analyzer and a Ti:sapphire oscillator that produces femtosecond pulses from 700 to 980 nm at 76 MHz repetition rate with pulse duration of ∼130 fs. The time resolution was 480 fs when the probe photon energy was set to 6.2 eV^56^. The infrared laser was frequency-quadrupled using BBO and KBBF crystals to produce an ultraviolet probe light from 177.5 to 230 nm. The beam sizes of pump and probe beam were set to ∼45 µm and ∼15 µm (full width at half maximum), respectively. The wavelengths of pump and probe beam were set to 800 and 200 nm, respectively. The repetition rate was set to 1000 kHz using a pulse picker. The energy and angular resolutions were set to be 8 meV and 0.1°, respectively. The Fermi level (*E*_F_) of the sample was calibrated with a gold film deposited onto the substrate. To avoid contamination of the sample surface in ex situ ARPES measurements, we covered the film with amorphous Se immediately after the epitaxy, transferred it to a separate ARPES chamber, and then decapped the amorphous Se film by heating under UHV.

STM measurements were carried out using a custom-made ultrahigh vacuum STM system^57^. Se capping layers for surface protection of TaSe_2_ films were removed in the STM chamber by Ar^+^ ion sputtering for 30 min and annealing at 200 °C for 60 min. STM measurements were performed with PtIr tips at 4.8 K under UHV below 2.0 × 10^−10^ Torr. All STM images were obtained in constant-current mode.

## Supplementary information

Supplementary Information

Peer Review File

## Data Availability

The data that support the findings of this study are available from the corresponding author upon reasonable request.
