# Supplementary material for: Robust charge-density wave strengthened by electron correlations in monolayer 1T-TaSe2 and 1T-NbSe2
Source: Nat Commun. 2021 Oct 7;12:5873. doi: 10.1038/s41467-021-26105-1 (PMC8497551; doi:10.1038/s41467-021-26105-1)
Supplement: Supplementary file 1 — Supplementary Information [file 41467_2021_26105_MOESM1_ESM.pdf]

SUPPLEMENTARY INFORMATION for

"Robust charge-density wave strengthened by electron correlations in monolayer 1T-$TaSe_2$ and 1T-$NbSe_2$" by Yuki Nakata *et al.*

Supplementary note 1: STM characterization of monolayer 1T-$TaSe_2$

We have performed scanning tunneling microscopy (STM) measurements on the monolayer 1T-$TaSe_2$ film grown on bilayer graphene substrate. As seen from the obtained STM image in a 100×100 nm^2 spatial region for the $TaSe_2$ film at T = 4. 8 K in Fig. S1a, a few triangular $TaSe_2$ islands (yellow region) are recognized on top of bilayer graphene substrate (dark region). We have also confirmed from the height profile along a cut across a step of $TaSe_2$ island in Fig. S1b (obtained along red arrow in Fig. S1a) that the step height is ~0.94 nm, which is in between monolayer (0.63 Å) and bilayer (1.26 Å) heights in bulk $TaSe_2$ [1], supporting the monolayer nature of our film.

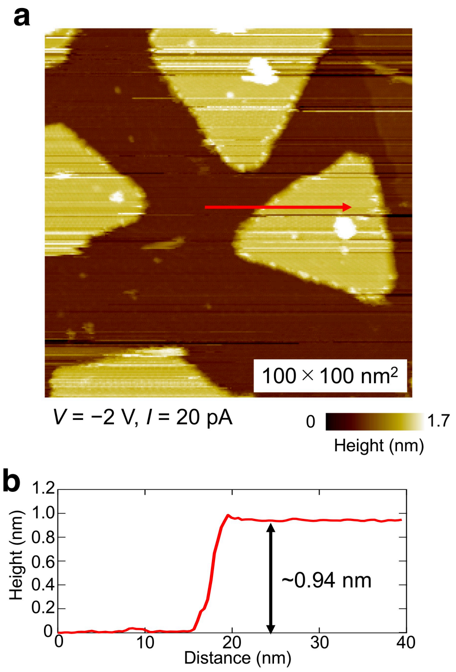

Supplementary Figure 1: STM characterization of monolayer 1T-$TaSe_2$. a, STM image in a surface area of 100×100 nm^2 for monolayer 1T-$TaSe_2$ on bilayer graphene measured at T = 4. 8 K. **b**, Height profile along a cut across a step of $TaSe_2$ island shown by red arrow in (a).

Supplementary note 2: Origin of insulating gap in monolayer 1T-$TaSe_2$

We discuss the origin of an insulating gap (Figs. 1-2) observed in the ARPES experiment of monolayer 1T-$TaSe_2$. Assuming that the observed gap is not a Mott-Hubbard gap, it could arise from any of the following four possibilities; (i) the Anderson gap, (ii) the band gap, (iii) the substrate-induced gap, and (iv) the conventional Fermi-surface-nesting-driven charge-density-wave (CDW) gap. The possibility (i) is ruled out because 1T-$TaSe_2$ is not a strongly disordered system and the experimental density of states (see e.g. Fig. 2d) does not show a power-law-like behavior expected from the disorder-induced Coulomb gap. The possibility (ii) is unlikely because the DFT (density functional theory) calculation of monolayer 1T-$TaSe_2$ neglecting U [2] was unable to reproduce a finite energy gap at the Γ point even when the energy position of the Fermi level (E_F) is intentionally moved up or down in the calculation to account for the possible self-doping of carriers to the film and/or the charge transfer from the substrate. The possibility (iii) is also unlikely because of the following reasons. If the gap opens due to the interaction with the substrate, the LHB of $TaSe_2$/$NbSe_2$ is expected to be hybridized with the graphene bands through a direct band overlap. However, this hybridization would not occur because the graphene band is 4 eV away from E_F around the Γ point. Lattice strain by the graphene substrate and resultant change in the band structure are unlikely to be responsible, because the lattice strain is expected to be weak due to the existence of van der Waals gap between $TaSe_2$/$NbSe_2$ and graphene, as suggested from the in-plane lattice constant estimated from the RHEED pattern in monolayer 1T-$TaSe_2$ (a = 3.5 Å [2]) which is similar to that of bulk (a = 3.47 Å [1]). One may think that the gap opening is due to the moiré potential associated with the lattice mismatch between $TaSe_2$/$NbSe_2$ and graphene. But this is also ruled out because the folded subband associated with the moiré potential is not observed. The possibility (iv) is also unlikely because the experiment does not support the emergence of any states within 0.3 eV of E_F

that are associated with the CDW-induced backfolded bands predicted in the calculation incorporating the superstructure potential with a √13×√13 periodicity (e.g. [3]). Also, a pseudogap, as observed in the nearly commensurate CCW (NCCDW) phase of bulk 1T-TaS_2 [4], is not seen. Based on these arguments, together with the fact that the GGA+U calculation [5] reasonably reproduces the overall experimental band dispersion, in particular, the gap opening at E_F, the observed gap is reasonably attributed to the electron-correlation-driven Mott-Hubbard gap (coexisting with CDW). This conclusion is also supported by the comparison of spectral feature between monolayer and bulk. As shown in Fig. S2a-S2c, overall ARPES intensity along the ΓM cut at low temperature appears to be similar between monolayer 1T-$TaSe_2$ and bulk samples of 1T-$TaSe_2$ and 1T-TaS_2 in which the Mott-Hubbard nature of the gap is well established. In particular, a nearly flat LHB around the Γ point, as well as the hybridization-gap discontinuity at $k \sim 2/3$ ΓM due to the formation of CDW, are commonly resolved in the ARPES intensity (Fig. S2a-S2c) and the corresponding plots of band dispersion (Fig. S2d-S2f) extracted from the peak position in EDCs.

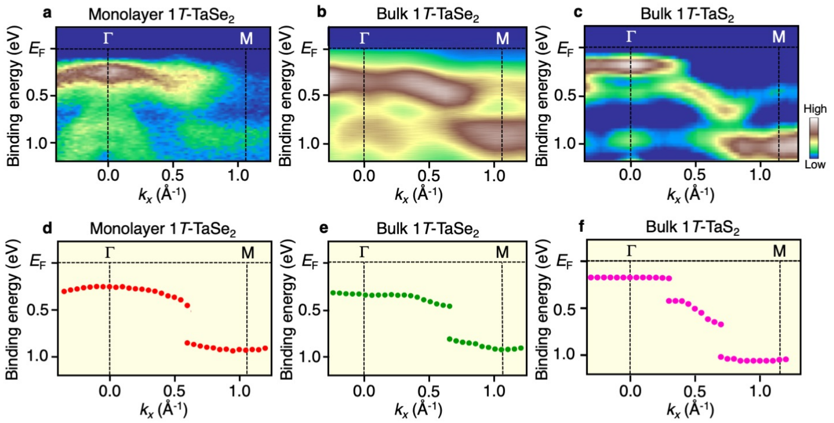

Supplementary Figure 2: Comparison of band dispersion among monolayer 1T-$TaSe_2$, bulk 1T-$TaSe_2$, and bulk 1T-TaS_2. a-c, ARPES intensity plots along the ΓM cut for monolayer 1T-$TaSe_2$, bulk 1T-$TaSe_2$, and bulk 1T-TaS_2, respectively, measured at $T = 30$-40 K. **d-f**, Corresponding experimental band dispersion extracted by tracking the peak position in EDCs.

Supplementary note 3: Comparison of ARPES and tunneling spectroscopy

Scanning tunneling microscopy/spectroscopy (STM/STS) experiments on monolayer 1T-$TaSe_2$ [6] reported (i) the local density of states (LDOS) that is asymmetric with respect to E_F, and (ii) an anticorrelated behavior in the dI/dV-conductance map between the lower and upper Hubbard bands (LHB and UHB). Thus, from the viewpoint of the LDOS and dI/dV maps, the electron-hole symmetry appears to be broken. The authors in ref. 6 have discussed that the anticorrelated behavior distinct from the expectation for an electron-hole symmetric Mott gap is still consistent with the Mott-gap picture and is due to additional effects associated with the tunneling process of electrons. Thus, the anticorrelation behavior in STM may not be against the Mott-insulator nature of the observed gap suggested by ARPES. In addition, the hybridization between the Hubbard bands and other bands may further enhance the particle-hole asymmetric behavior. It is known from previous STM studies that the anticorrelation becomes weak for multilayer (2-3 layer) 1T-$TaSe_2$ and bulk 1T-TaS_2 [7, 8]. This does not necessarily indicate that only monolayer sample exceptionally avoids the Mott-insulator behavior. It could rather be explained as: the CDW-Mott insulator behavior realized in monolayer becomes complicated in multilayer and bulk. Such complication may originate from the interlayer hopping, as inferred from the STM data on bulk 1T-TaS_2 displaying an intriguing modulation of LDOS around E_F depending strongly on the stacking sequence of David stars along the c-axis [8]. In this aspect, comparison of ARPES data between single- and multi-layers would be very interesting. Since the domain size of multilayer islands was found to be rather small, further optimization of epitaxial-growth condition and utilization of a small (submicron) beam spot are required to clarify the band structure of multilayer $TaSe_2$ films.

The previous STM study on monolayer 1T-$TaSe_2$ [6] has suggested the gap size of 0.11 eV by defining the width of energy region where the LDOS keeps the zero value, and this value is consistent with our *dI*/*dV* curve shown in Fig. S3. We have also estimated the Mott gap value called Δ_{Mott} from the energy position of LHB relative to E_F at the Γ point in the ARPES spectrum. We found that the energy position of LHB at Γ in the ARPES data nearly corresponds to the maximum of the valence-band peak in the tunneling spectrum in Fig. S3. We have estimated the energy interval between the maxima of valence and conduction bands in the tunneling spectrum to be ~0.5 eV. This value is consistent with the previous STM study [6] and about twice of the Δ_{Mott} value estimated from the ARPES data (0.56 eV), supporting the non-doped nature of our monolayer 1T-$TaSe_2$ film. Similarly, the non-doped condition is thought to be maintained for our monolayer 1T-$NbSe_2$ sample, as inferred from the previous STS measurement [9] showing that E_F is located at the center of full energy gap (zero density-of-states region).

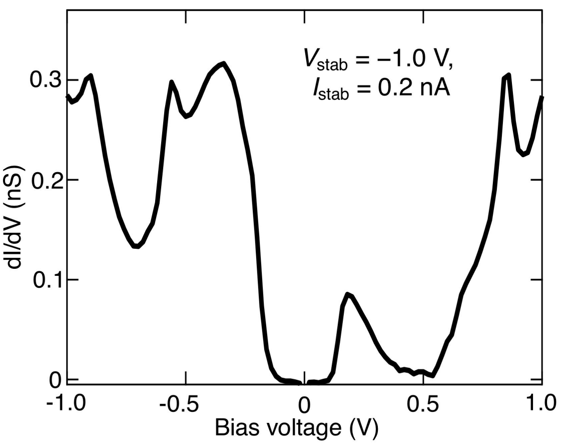

Supplementary Figure 3: Density of states of monolayer 1T-$TaSe_2$. Typical *dI*/*dV* spectrum on the monolayer $TaSe_2$ island measured at T = 4.8 K.

Since the zero-DOS region is clearly seen around E_F in the STM data for monolayer 1T-$TaSe_2$, it is expected that the electronic transport would also show insulating behavior. However, at the moment, it is difficult to obtain reliable transport data with our monolayer film, because the film is unstable in the atmosphere and therefore not suitable for performing *ex-situ* transport measurements. Even when we cover the sample with a protection layer, the electric current will selectively flow through the metallic bilayer graphene substrate (and the protection layer if it is conductive), and consequently, the insulating behavior of

monolayer film cannot be detected. *In-situ* transport measurements would be also difficult because of the same reason. Although the transport measurement is difficult, the zero-DOS behavior in the tunneling spectrum around the zero bias voltage strongly suggests the insulating nature of our monolayer 1T-$TaSe_2$ film.

Supplementary note 4: Temperature evolution of LHB

It is known from previous studies of bulk 1T-TaS_2 that the Mott and CDW states are coupled to each other. Such a coupling manifests as the NCCDW (nearly commensurate CDW) transition below T_{NCCDW} ~ 340 K associated with the formation of local David-star clusters, followed by a Mott transition (metal-insulator transition) at T_{Mott} ~ 200 K accompanying the CCDW (commensurate CCDW) with a $\sqrt{13}\times\sqrt{13}$ periodicity characterized by the fully stacked David-star clusters. Since the Mott transition is likely triggered by the band narrowing that follows the $\sqrt{13}\times\sqrt{13}$ superstructure potential, one can say that the necessary condition (but not the sufficient condition) of the Mott transition is the formation of CDW accompanying the David stars. It is known from the previous literatures [e.g. [4]] that one can distinguish (i) the normal state where both CDW and Mott states are absent, (ii) the Mott-insulator state, and (iii) the NCCDW state, by carefully inspecting the band structure. Specifically, (i) is characterized by the existence of a metallic band forming a large electron pocket centered at the M point as predicted from the first-principles band-structure calculations, (ii) is identified by the appearance of LHB with narrow bandwidth and opening of a full gap on entire Brillouin zone (BZ), and (iii) is confirmed by the absence of LHB and the recovery of nearly normal-state-like band structure with a pseudogap at the Fermi wave vectors (k_F's) on the electron pocket. Spectral distinction of these phases is known to be not so difficult. In particular, the Mott (CCDW) and NCCDW phases can be well distinguished because the transition between the two phases is accompanied by an abrupt change in the overall band

structure within the binding-energy range of ~1.2 eV [4].

Based on the above situation in bulk 1T-TaS_2, one can think about three possibilities in monolayer 1T-$TaSe_2$, i.e. (i) the CDW appears but the Mott state is not realized, and both CDW and Mott transitions take place (ii) at different temperatures ($T_{NCCDW} > T_{Mott}$) or (iii) at the same temperature ($T_{CDW} = T_{Mott}$). The case (i) is unlikely because we clearly observe a nearly flat band (the LHB) and a full gap on the entire BZ. The case (ii) is characterized by the disappearance of Mott gap and LHB as well as the appearance of CDW-originated pseudogap at $T_{Mott} < T < T_{NCCDW}$. What can be suggested from the present ARPES result is that a nearly flat band seen at 40 K still survives even at 450 K (Fig. 2c), suggestive of the persistence of LHB even at 450 K. This cannot be explained in terms of the absence of Mott gap and the persistence of simple CDW gap at 450 K because the spectral feature continuously evolves in the temperature range of 40 - 450 K, distinct from the abrupt change across T_{Mott} in bulk. Namely, whichever the case (ii) or case (iii) is realized, we can conclude that T_{Mott} for monolayer 1T-$TaSe_2$ is markedly enhanced compared to that for bulk 1T-TaS_2. Moreover, since T_{NCCDW} for bulk (~340 K) is much lower than 450 K, it is reasonable to conclude that the CDW transition temperature is also enhanced in monolayer.

We have examined whether or not the observed temperature dependence of the LHB can be explained with the simple thermal broadening effect. As shown in Fig. S4, we have tried to reproduce the EDC at T = 450 K (red curve) by intentionally broadening the EDC at T = 40 K with a gaussian, $\exp(-E^2/2\sigma^2)$, by varying its energy width σ (blue curve). When σ was chosen to match the slope of the leading edge (top curves), the spectral weight around the peak top is significantly overestimated in the simulation. On the other hand, when σ was chosen to match the overall EDC shape relatively well (bottom curves),

the simulated curve apparently has a broader leading edge. Also, it is noted that σ values used for the simulation [0.11 (0.18) eV which correspond to T = 1260 (2070) K for the top (bottom) EDC] are too large to be reconciled with a simple thermal effect. These results suggest that the observed temperature dependence of the LHB can hardly be explained with the simple thermal broadening picture, but reflects an intrinsic change in the gap magnitude.

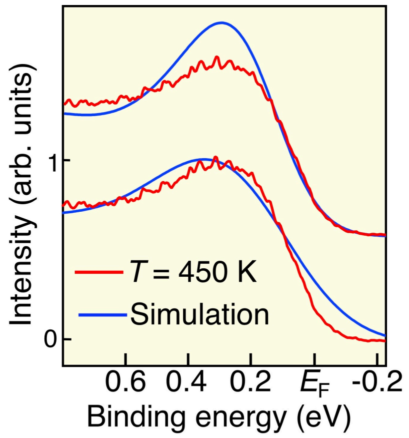

Supplementary Figure 4: Simulation of thermal broadening effect on LHB. EDC at the Γ point at T = 450 K (red curve) for 1ML 1T-$TaSe_2$ and simulated EDCs (blue curves) that were generated by broadening the experimental EDC at T = 40 K with a gaussian $\exp(-E^2/2\sigma^2)$ assuming σ = 0. 11 eV (top) and 0.18 eV (bottom).

Supplementary note 5: Difference between the leading-edge gap and spectroscopic gap

There exist two types of definitions in estimating the gap size from the EDC, i.e. (i) the energy position of the peak relative to E_F and (ii) the energy position of the leading-edge midpoint (LEM) relative to E_F. Both definitions are often used to discuss the gap size in other systems such as cuprates and Fe-based superconductors [10, 11]. In the present study, they are named Δ_{Mott} and Δ_{LEM}, respectively. We call Δ_{Mott} a spectroscopic gap, because $2\Delta_{Mott}$ spans the energy positions at which the LHB and UHB take the highest (and thereby spectroscopically prominent) density of states (DOS). The DOS for both the LHB and UHB is not so sharp and has a broad tail as can be seen from the ARPES data in Fig. 2d and tunneling spectrum in Fig. S3. Because the thermal excitation starts as soon as the excitation energy exceeds the zero DOS region around E_F, the transport measurement is sensitive to this tail. Since the definition of Δ_{LEM} inherently includes the tail region of the LHB, Δ_{LEM} is regarded to be sensitive to the transport gap (*i.e.* an activation gap in the transport measurements).

Supplementary note 6: Comparison of band structure between bulk and monolayer 1T-$TaSe_2$

We show in Fig. S5 the ARPES intensity plots along the ΓM cut for bulk 1T-$TaSe_2$ measured at T = (a) 30 K and (b) 300 K, compared with (c) that for monolayer 1T-$TaSe_2$ measured at T = 40 K (same as Fig. 4d in the main text). One may see from the comparison of Fig. S5a and S5c that the overall spectral intensity is roughly similar between bulk and monolayer at low temperature, e.g. in the existence of a hybridization-gap discontinuity at k ~ 2/3 ΓM due to the CDW formation. One can also see from the intensity plot at T = 300 K (b) that there exists no apparent band crossing of E_F and the spectral weight at E_F is still suppressed. In this regard, our result does not seem to be fully consistent with the previous study [12] which suggested the surface Mott transition occurring at T_{Mott} ~ 260 K. But this does not necessarily indicate that T_{Mott} at the surface of bulk 1T-$TaSe_2$ is far above room temperature (as high as that of monolayer), because even from the data for bulk at T = 30 K (Fig. S5a) one can clearly recognize a considerable intensity at E_F around the Γ point, in stark contrast to the almost zero intensity at E_F in monolayer (Fig. S5c). Such a crucial difference is highlighted by the comparison of low-temperature EDC at the Γ point in Fig. S5d; the result signifies a clear Fermi-edge cut-off in bulk (blue curve), as opposed to negligible spectral weight at E_F and steep intensity drop for the leading edge of LHB in monolayer (red curve).

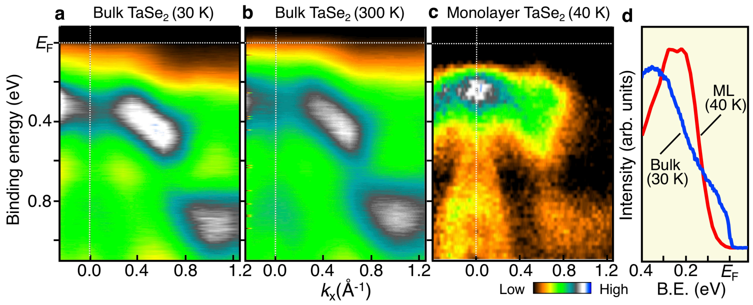

Supplementary Figure 5: Comparison of band dispersion between bulk and monolayer 1T-TaSe$_2$. a-c, ARPES intensity plots along the ΓM cut for bulk 1T-TaSe$_2$ measured at T = (**a**) 30 K and (**b**) 300 K, compared with (**c**) that for monolayer 1T-TaSe$_2$ measured at T = 40 K. **d**, EDC at the Γ point for bulk and monolayer 1T-TaSe$_2$, measured at T = 30 and 40 K, respectively.

Supplementary note 7: Spectral behavior in the photo-excited state

Figure S6a and S6b show the ARPES intensity around the Γ point measured with a 6.2-eV probe laser before (delay time, $t < 0$) and immediately after ($t = 0$) photo-excitation by a 1.55-eV pump laser, respectively. Taking into account the probe laser energy of $h\nu = 6.2$ eV and assuming that the work function of monolayer 1T-TaSe$_2$ is the same as that of bulk 1T-TaS$_2$ (5.2 eV) [13], one can roughly estimate the upper limit of photoelectron binding energy (E_B) to be 1 eV (= 6.2 – 5.2 eV). This E_B well covers the LHB located at ~0.3 eV. Also, a pump laser of $h\nu = 1.55$ eV enables the excitation of electrons to the UHB across the Mott gap of ~0.6 eV. Thus, our time-resolved ARPES measurement fully covers the important energy region involved for the Mott gap. It is noted that photoelectrons are detected at the low kinetic energy region outside the E_B range of 1 eV. Such a spurious photoelectron signal is known to appear because the low kinetic energy cut-off of photoelectron signal is usually broad unless a bias voltage is applied between the sample and analyzer. Also, it is known that photoelectrons with kinetic energy typically lower than a half of pass energy of the analyzer cannot be correctly measured by a standard ARPES apparatus.

As shown in Fig. S6a, at $t < 0$, one can clearly see peaks at $E_B \sim 0.3$, 0.55, and 0.8 eV, which are attributed to the LHB, and two Se-4*p* bands, respectively, by referring to the experimental result in Fig. 2a measured with the He-Iα line ($h\nu = 21.218$ eV). While the intensity of LHB is significantly suppressed because of the marked reduction of the Ta-5*d*/Se-4*p* photoionization cross-section ratio [14], one can still recognize in Fig. S6a a broad peak corresponding to the LHB, as better visualized in the EDC at the Γ point in Fig. S6d (blue curve). After photo-excitation (Fig. S6b), the overall spectral feature becomes broad, but one can still see a weak but finite LHB intensity. This is supported by appearance of a broad hump at $E_B \sim 0.3$ eV in the EDC in Fig. S6d (red curve), followed by the Se 4*p* main peak at $E_B \sim 0.5$ eV.

Considering the high pump fluence to trigger the electronic phase transition in bulk Ta-based TMDs [15-20], we have tried to increase the pump fluence as much as possible, and found that 0.26 mJ/cm^2 is an upper limit for reliable measurements, above which an

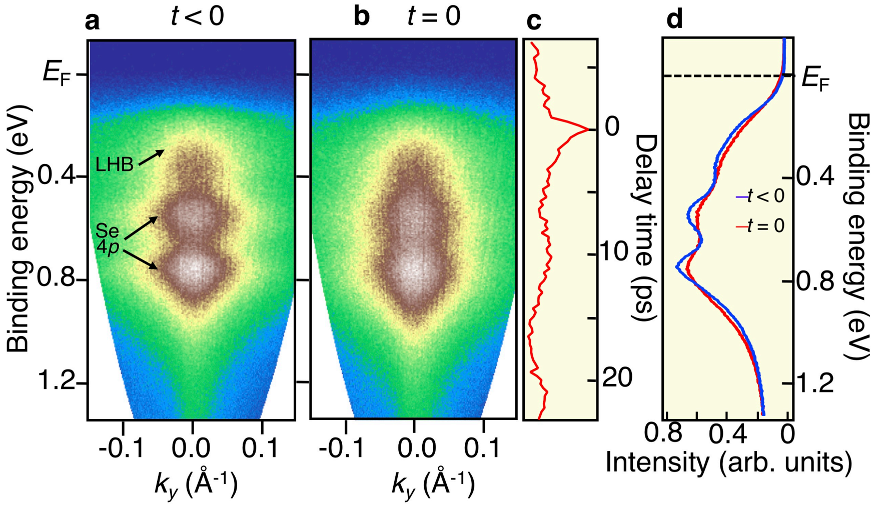

Supplementary Figure 6: Time-resolved ARPES measurements for monolayer 1T-TaSe$_2$. **a** and **b**, ARPES intensity around the Γ point at $T = 80$ K measured with a 6.2-eV probe laser before (delay time, $t < 0$) and immediately after ($t = 0$) photo-excitation by a pump laser of 1.55 eV, respectively. **c**, Delay-time dependence of photoelectron intensity at $E_B = 0.314$ eV (the E_B position of LHB). We set the pump laser power to be as high as possible (0.26 mJ/cm^2), above which the monolayer sample showed a rapid and irreversible degradation. **d**, EDCs at the Γ point before ($t < 0$; blue curve) and immediately after ($t = 0$; red curve) photo-excitation.

irreversible spectral broadening was observed due to the photo-induced damage of the monolayer sample. Therefore, we carried out the time-resolved ARPES measurement with 0.26 mJ/cm^2 pump fluence. On the other hand, there exist some reports that bulk samples are still robust at this pump fluence because the pump fluence higher than 1 mJ/cm^2 could be applied without damaging the sample [15-20]. This suggests that the monolayer sample is structurally and/or chemically more fragile against the pump laser irradiation than the bulk counterpart. This may be related to the absence of interlayer coupling in monolayer which may help strengthen the overall stability of sample. Thus, direct comparison of the robustness of CDW-Mott phase against photo-excitations between monolayer and bulk under identical experimental condition is difficult at the moment.

Supplementary note 8: Geometry of ARPES measurements

ARPES data shown in Fig. 3 were obtained by using linearly polarized photons from synchrotron radiation while those in Figs. 1 (except for the data in Fig. 1f which was measured with circularly polarized 260-eV photons), 2, and 4 were recorded with the linearly polarized He-Iα photons in laboratory (linear polarization is due to grating). Both data were obtained using the “angular mode” of electron analyzer which simultaneously collects photoelectrons with a finite acceptance angle along the *y* direction, as shown in Fig. S7. In the case of laboratory-based measurement (Fig. S7a), the light is in *x*-*z* plane,

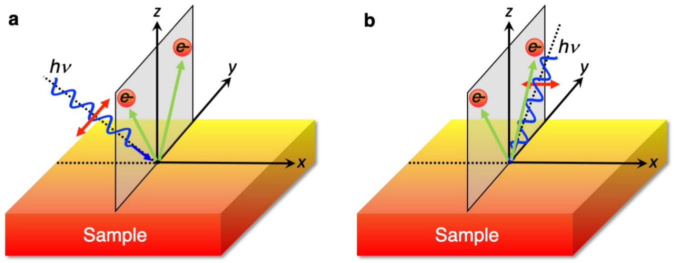

Supplementary Figure 7: Schematics of experimental geometry for ARPES measurements. Experimental geometry of incident light, emitted photoelectrons, and sample surface for the **a**, laboratory-based and **b**, synchrotron-based ARPES measurements.

and the geometry is equivalent for photoelectrons emitted to the positive vs negative *y* directions. On the other hand, as shown in Fig. S7b, the light is in *y*-*z* plane in the synchrotron-based measurement, and it is inequivalent. This causes asymmetry in the photoelectron matrix-element effect between positive and negative k_y's, as seen in Fig. 2a of main text. It is noted that the intensity asymmetry does not affect the main conclusion of the present study, because the matrix-element effect does not influence the energy position of bands.

Supplementary references

1. Wilson, J. A., Di Salvo, F. J. & Mahajan, S. Charge-density waves and superlattices in the metallic layered transition metal dichalcogenides. *Adv. Phys.* **24**, 117-201 (1975).
2. Nakata, Y. *et al.* Selective fabrication of Mott-insulating and metallic monolayer $TaSe_2$. *ACS Appl. Nano Mater.* **1**, 1456-1460 (2018).
3. Sohrt, C., Stange, A., Bauer, M., & Rossnagel, K. How fast can a Peierls–Mott insulator be melted? *Faraday Discuss.* **171**, 243-257 (2014).
4. Ang, R. *et al.* Real-space coexistence of the melted Mott state and superconductivity in Fe-substituted 1T-TaS_2. *Phys. Rev. Lett.* **109**, 176403 (2012).
5. Calandra, M. Phonon-assisted magnetic Mott-insulating state in the charge density wave Phase of Single-Layer 1T-$NbSe_2$. *Phys. Rev. Lett.* **121**, 026401 (2018).
6. Chen, Y. *et al.* Strong correlations and orbital texture in single-layer 1T-$TaSe_2$. *Nat. Phys.* **16**, 218-224 (2020).
7. Qiao, S. *et al.* Mottness collapse in 1T-$TaS_{2-x}Se_x$ transition-metal dichalcogenide: an interplay between localized and itinerant orbitals. *Phys. Rev. X* **7**, 041054 (2017).
8. Butler, C. J., Yoshida, M., Hanaguri, T., & Iwasa, Y. Mottness versus unit-cell doubling as the driver of the insulating state in 1T-TaS_2. Nature Commun. **11**, 2477 (2020).
9. Nakata, Y. *et al.* Monolayer 1T-$NbSe_2$ as a Mott insulator. *NPG Asia Mater.* **8**, e321 (2016).
10. Ding, H. *et al.* Spectroscopic evidence for a pseudogap in the normal state of underdoped high-Tc superconductors. *Nature* **382**, 51-54 (1996).

11. Kanigel, A. *et al*. Evolution of the pseudogap from Fermi arcs to the nodal liquid. *Nat. Phys*. **2**, 447 (2006).
12. Perfetti, L. *et al*. Spectroscopic signatures of a bandwidth-controlled Mott transition at the surface of 1T-$TaSe_2$. *Phys. Rev. Lett.* **90**, 166401 (2003).
13. Shimada, T., Ohuchi, F. S., & Parkinson, B. A. Work function and photothreshold of layered metal dichalcogenides. *Jpn. J. Appl. Phys.* **33**, 2696 (1994).
14. Yeh, J. J. & Lindau, I. *ATOMIC DATA AND NUCLEAR DATA TABLES.* **32**, l-l55 (1985).
15. Perfetti, L. *et al*. Femtosecond dynamics of electronic states in the Mott insulator 1T-TaS_2 by time resolved photoelectron spectroscopy. *New J. Phys.* **10**, 053019 (2008).
16. Perfetti, L. *et al*. Time evolution of the electronic structure of 1T-TaS_2 through the insulator-metal transition. *Phys. Rev. Lett.* **97**, 067402 (2006).
17. Sohrt, C., Stange, A., Bauer, M., & Rossnagel, K. How fast can a Peierls–Mott insulator be melted? *Faraday Discuss.* **171**, 243-257 (2014).
18. Vaskivskyi, I. *et al*. Controlling the metal-to-insulator relaxation of the metastable hidden quantum state in 1T-TaS_2. *Sci. Adv.* **1**, e1500168 (2015).
19. Shi, X. *et al.* Ultrafast electron calorimetry uncovers a new long-lived metastable state in 1T-$TaSe_2$ mediated by mode-selective electron-phonon coupling. *Sci. Adv.* **5**, eaav4449 (2019).
20. Zhang, Y. *et al*. Coherent modulation of the electron temperature and electron–phonon couplings in a 2D material. *Proc. Natl. Acad. Sci., USA* **117**, 8788 (2020).
